# Physical performance capacity after pediatric kidney transplant and clinical parameters associated with physical performance capacity

**DOI:** 10.1007/s00467-022-05758-0

**Published:** 2022-10-31

**Authors:** Heidi Mäenpää, Juuso Tainio, Jari Arokoski, Timo Jahnukainen

**Affiliations:** 1 Department of Rehabilitation, New Children’s Hospital, Helsinki University Hospital, PO Box 347, 00029, HUS Helsinki, Finland; 2 University of Helsinki, Helsinki, Finland; 3 Department of Pediatric Nephrology and Transplantation, New Children’s Hospital, Helsinki University Hospital, Helsinki, Finland; 4 Department of Physical and Rehabilitation Medicine, Helsinki University Hospital, Helsinki, Finland

**Keywords:** Pediatric kidney transplant recipient, Physical performance capacity, Congenital nephrosis of Finnish type, Clinical parameters

## Abstract

**Background:**

History of chronic kidney disease and kidney transplantation is known to influence physical performance capacity. The aim of this study was to compare the physical performance of pediatric kidney transplant recipients to healthy controls and to find possible correlations between clinical parameters and physical performance capacity.

**Methods:**

Twenty-four pediatric kidney transplant recipients (62.5% boys) were tested at a median age of 10.8 years. Physical performance capacity was tested with a test set including six different components assessing muscle endurance, strength, speed, and flexibility. The control group consisted of 273 healthy age-matched schoolchildren. Clinical parameters were collected as part of routine follow-up protocol. The majority of patients (62.5%) had congenital nephrotic syndrome of Finnish type (CNS) as primary diagnosis, and therefore, the results of CNS recipients were compared to the other disease groups.

**Results:**

The physical performance capacity in pediatric kidney transplant recipients was lower compared to healthy controls. Surprisingly, no statistically significant correlation was found between graft function and physical performance capacity. The CNS patients scored worse than patients with other diagnoses in all test domains except for sit-and-reach and shuttle run, but the differences did not reach statistical significance.

**Conclusion:**

The physical performance of pediatric kidney transplant recipients is reduced, especially in those with congenital nephrotic syndrome. Clinical parameters, including graft function, did not predict physical performance capacity, suggesting that the reduced physical performance seems to be of multivariable cause.

**Graphical abstract:**

**Figure:**
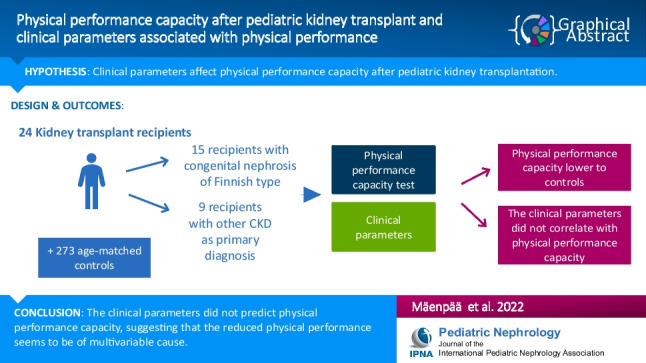
A higher resolution version of the Graphical abstract is available as Supplementary information

**Supplementary Information:**

The online version contains supplementary material available at 10.1007/s00467-022-05758-0.

## Introduction

Kidney transplantation has become the preferred treatment in pediatric severe chronic kidney disease (CKD). With 10-year patient survival rate exceeding 90% [1], the care of this population is now being challenged by treatment-related long-term outcomes involving metabolic and cardiovascular diseases, decreased exercise capacity, and sedentary lifestyle.

We, among others, have shown that the physical performance of pediatric patients with a history of solid organ transplantation is decreased [2]. Muscle deficits and altered muscle metabolism affect pediatric patients with CKD [3, 4], although Matsumoto et al. [5] demonstrated that the abnormalities in the oxidative metabolism of muscles in children with severe kidney failure are reversed with successful kidney transplantation. The reason for the diminished physical activity, and therefore probably also for the decreased fitness, is likely to be multifactorial, including previous CKD, fatigue, frailty, not being used to exercise, fear of kidney allograft injury, having received advice to limit physical activity, and insufficient exercise guidelines [6–9].

Exercise capacity and its associations with clinical parameters after kidney transplantation are of clinical interest. In pediatric recipients, no correlations have been found between dialysis duration, time since transplantation, kidney function estimated by glomerular filtration rate (GFR), BMI, blood pressure medication, hematocrit or serum phosphorus levels, and exercise capacity [10–13]. There are only few studies addressing physical performance and its association with clinical parameters in pediatric kidney transplant recipients, and the results are somewhat controversial. We therefore aimed to study in this nationwide study the physical performance of pediatric kidney transplant recipients in comparison to age- and gender-matched control subjects.

Approximately 40% of pediatric kidney transplants in Finland are performed for patients with congenital nephrosis of the Finnish type (CNF). Long-lasting hypoproteinemia before nephrectomy is known to lead to muscle hypotonia. We therefore compared physical performance between CNS patients and other kidney transplant recipients to see whether there are any differences between the two groups. We hypothesized that physical performance would be inferior in pediatric kidney transplant recipients when compared to controls, as previously stated [2]. We also assumed that at least measured GFR (mGFR) and BMI would correlate with physical performance.

## Materials and methods

### Kidney transplant recipients

All pediatric kidney transplants in Finland are performed at the study center and the recipients are followed up at our unit at least yearly. We enrolled recipients to the study during their annual follow-up visit with the following inclusion criteria: (1) history of kidney transplantation, (2) age 10–12 years, and (3) capability to perform the tests used. A total of 41 eligible recipients were identified; 5 of them were excluded because of medical reasons (neurology (*n* = 3), recent transplantation (*n* = 1), and recent scoliosis operation (*n* = 1)). In addition, 12 recipients did not participate in the study due to other reasons, such as scheduling or logistic problems. This study cohort included 19 recipients from our previous study [2] with the addition of five previously unassessed recipients. These five recipients were added to increase the number of recipients in the study and because the testing continued as a routine physiotherapy check-up in our follow-up protocol.

Finally, 24/41 (58.5%) of the eligible recipients were enrolled between November 2016 and July 2021, including one child aged 8.1 and another aged 13.4 years, since they were already tested, to increase the number of recipients in the study. The pre-transplant diagnoses were as follows: congenital anomalies of the kidney and the urinary tract (CAKUT) (*n* = 5), congenital nephrotic syndrome of the Finnish type (CNS) (*n* = 15), and acquired diseases, including hemolytic uremic syndrome, acute kidney injury, CKD unspecified, and congenital kidney failure (*n* = 4).

The study was approved by the Ethics Committee of the Helsinki University Hospital. An information leaflet was given to all participants and their guardians, and all patients and parents approved participation in the study.

### Control subjects

The age- and gender-matched control group consisted of 273 school children tested in primary and secondary school. These children were previously enrolled and described in more detail in a study evaluating physical performance in pediatric autologous stem cell transplant recipients [14].

### Tests for physical performance

The recipients were tested during their routine yearly checkups at the study center. All tests were performed by trained pediatric physiotherapists. Recipients were asked about their physical activity, i.e., sports hobbies and attending physical exercise (PE) at school, and this was described in medical records.

Physical performance was evaluated using a standardized test set, which has been described in our recent study [2], as well as in studies on the outcomes of pediatric autologous stem cell transplantation [14] and non-transplanted childhood leukemia survivors [15]. The tests were partly modified from those generally used in healthy subjects, such as Eurofit [16, 17]. The set included six different tests: leg-lift and repeated squatting test (for 30 s), measuring lower body muscle endurance and strength, and sit-up and back extension test (for 30 s), measuring core muscle endurance and strength. These four tests were recorded by repetitions per 30 s. Flexibility was tested with sit-and-reach test and measured in centimeters. Speed, acceleration, and agility were tested with shuttle run and measured by time (seconds). A higher score indicates better results in all the tests, except for shuttle run, in which a lower score means a faster (better) result. The shuttle run test was not performed for a significant subset of patients (7/24) due to problems with slipperiness of the floor.

The key clinical data of the recipients were obtained from medical reports. These test results were obtained simultaneously with the physical performance testing. The data included a wide range of laboratory results, height, weight, and systolic and diastolic blood pressure. These data were measured as part of the routine follow-up protocol. The neurological and psychological status of all the recipients was obtained retrospectively from the records and labeled as normal/deviant. All recipients were evaluated by a pediatric neurologist and psychiatrist as per the routine pre-transplant protocol and followed up according to the results. All recipients underwent a neuropsychological evaluation at preschool age. Early motor development was obtained from physiotherapy records and labeled as normal/deviant/delayed regarding the achievement of early motor milestones. All patients received triple-drug immunosuppression including calcineurin inhibitor (ciclosporin A or tacrolimus), antimetabolite (azathioprine or mycophenolate), and methylprednisolone. Rejections were detected either at protocol or indication biopsies and classified according to the Banff classification [18]. Laboratory parameters were not available from the healthy control subjects.

Office and ambulatory blood pressures (BP) were measured during each annual follow-up visit or at a preceding outpatient visit within 3 months of the physical performance test. Office BP was measured repeatedly at minimum three times and ambulatory BP was by an oscillometric monitor (ABPM 5100 or ABPM 6100, Welch Allyn Inc., Skaneateles Falls, NY, USA) or Schiller BR-102 Plus (Schiller AG, Baar, Switzerland). BP was categorized as normal if below the 90th percentile and hypertensive if equal to or above the 95th percentile [19]. Forty-six percent of the studied patients were on antihypertensive medication (amlodipine 10/24 and enalapril 1/24).

### Statistical methods

All statistical analyses were performed using IBM SPSS Statistics 27 (SPSS Inc., Chicago, IL, USA). The results are presented as mean (SD) or median (range) or number of recipients (%), as appropriate. Comparisons of the groups were performed with the Mann–Whitney *U*-test and correlations were analyzed with Spearman’s correlation coefficient.

All physical test results were indexed to remove the effect of age or gender. The indexes were calculated by dividing the physical test results with the mean result of control children by age and gender. A physical performance capacity total score was calculated by adding together all indexed scores of an individual’s results, except for shuttle run due to the relatively large number of missing performances.

## Results

### Recipient demographics

The median age at the time of the study was 10.8 (8.1–13.4) years. The median age at the time of the transplantation was 1.5 (1.0–13.2) years and the minimal time since transplantation was 2.6 months. There were more males (62.5%) than females in the study cohort (Table 1). Fifteen (62.5%) of the recipients had CNS as primary diagnosis. Recipients with CNS were lighter (32.2 vs. 42.4, *p* = 0.02) and their BMI was lower (16.9 vs. 20.1, *p* = 0.01) compared to the other recipients at the time of the study. Patients with CNS more often had peritoneal dialysis as treatment modality (*p* = 0.05) compared to patients with other diagnoses. The duration of dialysis did not, however, differ significantly between the diagnosis groups (*p* = 0.60). We found no significant differences in any other recipient characteristics.

**Table 1:** Patient characteristics in 24 pediatric kidney transplant recipients

	KTx (*N* = 24)
Diagnoses, number of recipients (%)	
Hemolytic uremic syndrome	1 (4.2)
Finnish-type congenital nephrosis	15 (62.5)
Acute kidney failure	1 (4.2)
Chronic kidney disease, unspecified	1 (4.2)
Congenital kidney failure	1 (4.2)
Congenital posterior urethral valves	3 (12.5)
Congenital malformations of the urinary system	1 (4.2)
Congenital malformation of the urinary system, unspecified	1 (4.2)
Median age at the time of testing, years	10.79 (8.1–13.4)
Median age at the time of transplantation, years	1.54 (1.0–13.2)
Median time since transplantation, months	108.2 (2.6–132.9)
Male gender, number of recipients (%)	15 (62.5%)
Systolic blood pressure (mmHg)	112.0 (87.0–129.0)
Diastolic blood pressure (mmHg)	73.0 (52.0–88.0)
Dialysis treatment modality, number of recipients (%)	
Peritoneal dialysis	16 (66.7%)
Hemodialysis	3 (12.5%)
Peritoneal dialysis and hemodialysis	2 (8.3%)
No dialysis treatment	3 (12.5%)
Duration of dialysis, days	280 (0–748)
Number of rejections	0.0 (0–2)
Laboratory results	
mGFR (mL/min/1.73 m^2^)	49.0 (29.0–130.0)
Creatinine (μmol/L)	88.5 (32–209)
Cystatin C (mg/L)	1.67 (0.9–3.2)
Urea (mmol/L)	11.2 (4.1–19.4)
Hemoglobin (g/L)	119.5 (97.0–147.0)
Potassium (mmol/L)	4.3 (2.8–5.8)
Sodium (mmol/L)	141.0 (136.0–145.0)
Phosphate (mmol/L)	1.36 (1.0–1.8)
Magnesium (mmol/L)	0.75 (0.5–0.9)
Calcium-ionized (mmol/L/pH 7.4)	1.23 (1.2–1.3)
PTH (ng/L)	71.0 (29–161)^a^
FPG (mmol/L)	5.4 (4.6–7.5)^b^
HbA1c (mmol/mmol)	35.5 (32.0–39.0)^c^
Triglyceride (mmol/L)	0.96 (0.5–3.1)^d^
ESR (mm/h)	7.5 (2–60)^d^
CRP (mg/L)	3.0 (3–4)
Urine protein (mg/L)	105.0 (45–1330)^e^

Values are median (range) unless otherwise stated. FPG and HbA1c values within 3 years of the physical test date

KTx, kidney transplantation; mGFR, measured glomerular filtration rate; PTH, parathyroid hormone; FPG, fasting plasma glucose; ESR, erythrocyte sedimentation rate; CRP, C-reactive protein

^a^Data missing in 1 recipient

^b^Data missing in 13 recipients

^c^Data missing in 14 recipients

^d^Data missing in 2 recipients

^e^Data missing in 5 recipients

### Physical performance of controls

The median age at testing was 11.0 (8.0–13.0) years. The controls were taller (146.2 vs. 140.0, *p* = 0.03) and weighed more (40.7 vs. 36.1, *p* = 0.06) than the recipients. The controls performed better than the recipients in all test domains except in sit-and-reach test. In leg lift test, sit-and-reach test and shuttle run test the controls achieved indexed mean results of the age group.

### Physical performance and clinical parameters in recipients

In line with our previous study [2], in this patient cohort, including an additional five patients, the kidney transplant recipients performed significantly worse than the controls in sit-up test, back extension test, and shuttle run test (Table 2). The kidney transplant recipients performed statistically significantly better than the controls in sit-and-reach test. In leg lift and repeated squatting test, the kidney transplant recipients performed not significantly different from the controls (Table 2).

**Table 2:** Patient and control comparisons in height, weight, body mass index, and indexed physical test results

	Kidney Tx (*N* = 24)	Control (*N* = 273)	*p*-Value
Age at the time of testing, years	11.08 (1.2)	10.68 (1.8)	0.30
Height, cm	140.0 (10.9)	146.2 (12.7)	0.03
Weight, kg	36.1 (10.2)	40.7 (12.4)	0.06
BMI	18.08 (2.9)	18.65 (3.3)	0.05
Physical test results			
Leg lift, repetitions	0.98 (0.1)	1.0 (0.1)	0.33
Repeated squatting test, repetitions	0.97 (0.1)	0.99 (0.1)	0.74
Sit-up test, repetitions	0.66 (0.4)	0.97 (0.3)	0.00
Sit-and-reach test, cm	1.07 (0.1)	1.0 (0.1)	0.00
Back extension test, repetitions	0.60 (0.3)	0.99 (0.1)	0.00
Shuttle run test, s	1.15 (0.2)^a^	1.01 (0.1)	0.01
Physical performance capacity^**^	4.26 (0.8)	4.95 (0.6)	0.00

Results presented as mean (SD). Mann–Whitney *U* test was used in data-analysis

BMI, body mass index; Tx, transplantation

^a^Data missing in 7 recipients

^**^Physical performance capacity was computed by adding together all physical test (except shuttle run) scores

When comparing the association of clinical parameters with physical capacity performance, we found a statistically significant, yet moderate correlation with plasma magnesium level (P-Mg) and Physical Performance Total Score (0.42, *p* = 0.04) (Table 3), height and sit-and-reach test (− 0.43, *p* = 0.04), diastolic blood pressure and sit-up test (0.67, *p* = 0.00), P-Mg and back extension (0.56, *p* = 0.00), potassium and sit-up (− 0.46, *p* = 0.03), dialysis duration and leg lift (− 0.47, *p* = 0.02), neurology and leg lift (− 0.57, *p* = 0.00), repeated squatting (− 0.49, *p* = 0.02), and shuttle run (0.57, *p* = 0.02) and fasting plasma glucose (FPG) and shuttle run (0.69, *p* = 0.04) (Table 4). mGFR (0.15, *p* = 0.5) or BMI (− 0.09, *p* = 0.68) did not correlate with physical activity in this cohort (Table 3).

**Table 3:** Correlation between indexed Physical Performance Capacity Total Score and recipient characteristics in 24 pediatric kidney transplant patients

	*r*_*s*_	*p*-Value
Median age at the time of testing	0.25	0.25
Median age at the time of transplant	− 0.04	0.85
Height	0.06	0.78
Weight	− 0.04	0.84
BMI	− 0.09	0.68
Systolic blood pressure (mmHg)	0.36	0.10
Diastolic blood pressure (mmHg)	0.41	0.06
Number of rejections	− 0.13	0.56
Dialysis duration	− 0.38	0.07
Dialysis treatment modality	0.27	0.21
Neurology (normal/deviant)	− 0.37	0.07
Psychic challenges (normal/deviant)	0.10	0.65
Laboratory results		
mGFR (mL/min/1.73 m^2^)	0.15	0.50
Creatinine (μmol/L)	0.09	0.67
Cystatin C (mg/L)	0.01	0.96
Urea (mmol/L)	− 0.27	0.20
Hemoglobin (g/L)	0.26	0.22
Potassium (mmol/L)	− 0.33	0.12
Sodium (mmol/L)	− 0.28	0.19
Phosphate (mmol/L)	− 0.03	0.87
Magnesium (mmol/L)	0.42	0.04
Calcium-ionized (mmol/L/pH 7.4)	− 0.15	0.49
PTH (ng/L)	0.31	0.15
FPG (mmol/L)	− 0.30^a^	0.38
HbA1c (mmol/mmol)	− 0.01^b^	0.97
Triglyceride (mmol/L)	− 0.05	0.83
ESR (mm/h)	− 0.06	0.81
CRP (mg/L)	0.29	0.17
Urine protein (mg/L)	− 0.03	0.91

Spearman’s correlation coefficient (*r*_*s*_) was used for data analyses, *p*-value two-tailed. Indexing was performed by dividing the physical test results with the mean result by age and gender. FPG and HbA1c values within 3 years of the physical test date

mGFR, measured glomerular filtration rate; PTH, parathyroid hormone; FPG, fasting plasma glucose; ESR, erythrocyte sedimentation rate; CRP, C-reactive protein

^a^Data missing in 13 recipients

^b^Data missing in 14 recipients

**Table 4:** Correlation coefficient of indexed physical test results and recipients characteristics in 24 pediatric kidney transplant recipients

	Leg lift		Repeated squatting		Sit-up		Sit-and-reach		Back extension		Shuttle run	
	r_s_	*p*-value	r_s_	*p*-value	r_s_	*p*-value	r_s_	*p*-value	r_s_	*p*-value	r_s_	*p*-value
Median age at the time of testing	0.26	0.23	0.07	0.76	0.13	0.55	–0.27	0.20	0.15	0.49	–0.05 ^d^	0.84
Median age at the time of transplant	0.03	0.88	0.06	0.78	–0.13	0.53	–0.12	0.56	0.10	0.66	–0.46 ^d^	0.06
Height	–0.03	0.88	–0.19	0.38	0.06	0.78	–0.43	0.04*	0.02	0.92	0.05 ^d^	0.84
Weight	–0.01	0.97	–0.12	0.58	–0.06	0.80	–0.32	0.13	–0.11	0.63	–0.01 ^d^	0.96
BMI	0.05	0.83	0.09	0.68	–0.19	0.37	–0.08	0.72	–0.04	0.86	–0.09 ^d^	0.74
Systolic blood pressure (mmHg)	0.00	0.99	0.01 ^b^	0.96	0.29 ^b^	0.19	0.20 ^b^	0.36	0.38 ^b^	0.08	–0.22 ^e^	0.42
Diastolic blood pressure (mmHg)	0.21	0.36	0.13 ^b^	0.58	0.67 ^b^	0.00*	0.36 ^b^	0.10	0.31 ^b^	0.16	0.28 ^e^	0.29
Number of rejections	0.04	0.86	0.02	0.94	–0.19	0.38	0.16	0.47	0.05	0.83	–0.17 ^d^	0.52
Dialysis duration	–0.47	0.02*	–0.24	0.27	–0.37	0.08	0.19	0.38	–0.21	0.32	0.31	0.23
Dialysis treatment modality	0.14	0.51	0.06	0.80	0.11	0.62	0.22	0.32	0.16	0.48	0.26^e^	0.31
Neurology (normal/deviant)	–0.57	0.00*	–0.49	0.02*	–0.25	0.23	0.32	0.12	–0.34	0.10	0.57	0.02*
Psychic challenges (normal/deviant)	0.34	0.10	–0.04	0.86	–0.12	0.58	–0.26	0.22	–0.22	0.31	–0.47 ^d^	0.06
Laboratory results												
mGFR (mL/min/1.73 m^2^)	0.20	0.36	0.13	0.55	0.11	0.60	–0.12	0.56	0.04	0.84	0.15 ^d^	0.56
Creatinine (umol/L)	0.01	0.95	–0.04	0.84	0.18	0.39	0.03	0.91	0.11	0.62	0.11 ^d^	0.67
Cystatin C (mg/L)	–0.06	0.77	0.02	0.91	–0.05	0.81	0.31	0.14	0.11	0.61	0.04 ^d^	0.87
Urea (mmol/L)	–0.31	0.14	–0.33	0.12	–0.22	0.30	0.09	0.68	–0.26	0.22	–0.16 ^d^	0.55
Hemoglobin (g/L)	–0.02	0.92	0.18	0.39	0.17	0.43	–0.13	0.54	0.34	0.11	–0.16 ^d^	0.54
Urine protein (mg/L)	–0.42 ^c^	0.08	0.39 ^c^	0.10	0.07 ^c^	0.79	0.06 ^c^	0.79	–0.01 ^c^	0.97	–0.45 ^g^	0.11
Potassium (mmol/L)	–0.16	0.45	–0.18	0.40	–0.46	0.03*	0.12	0.58	–0.30	0.15	–0.07 ^d^	0.79
Sodium (mmol/L)	0.03	0.89	–0.26	0.21	–0.26	0.22	–0.15	0.50	–0.25	0.24	0.22 ^d^	0.40
Phosphate (mmol/L)	0.10	0.64	0.19	0.38	–0.26	0.22	0.08	0.70	0.19	0.38	0.05 ^d^	0.86
Magnesium (mmol/L)	0.33	0.12	0.33	0.12	0.11	0.60	0.35	0.09	0.56	0.00*	0.18 ^d^	0.50
Calcium-ionized (mmol/L/pH 7.4)	–0.16	0.46	–0.34	0.10	0.02	0.92	–0.18	0.40	–0.17	0.43	–0.27 ^d^	0.29
PTH (ng/L)	0.11 ^a^	0.63	0.03 ^a^	0.91	0.34 ^a^	0.12	0.15 ^a^	0.51	0.31 ^a^	0.15	–0.00 ^e^	1.00
FPG (mmol/L)	–0.22 ^i^	0.53	–0.01 ^i^	0.98	–0.35 ^i^	0.29	–0.11 ^i^	0.74	–0.21 ^i^	0.54	0.69 ^h^	0.04*
HbA1c (mmol/mmol)	–0.04 ^j^	0.90	0.49 ^j^	0.15	–0.41 ^j^	0.24	0.20 ^j^	0.58	–0.28 ^j^	0.43	–0.11 ^k^	0.80
Vitamin D	–0.03 ^a^	0.88	–0.11 ^a^	0.63	–0.15 ^a^	0.49	–0.12 ^a^	0.60	–0.30 ^a^	0.17	0.15 ^e^	0.57
Triglyceride (mmol/L)	0.08	0.74	–0.23 ^b^	0.30	0.04 ^b^	0.86	–0.22 ^b^	0.32	–0.03 ^b^	0.88	–0.41 ^f^	0.13
ESR (mm/i)	–0.05 ^b^	0.82	–0.01 ^b^	0.98	–0.16 ^b^	0.47	–0.21 ^b^	0.34	0.27 ^b^	0.22	0.01 ^e^	0.98
CRP (mg/L)	–0.02 ^b^	0.95	0.18	0.40	0.29	0.18	0.29	0.17	0.21	0.33	0.40 ^d^	0.12

r□=Spearman´s correlation coefficient was used, *p*-value two-tailed, ^a^Data missing in 1 recipient, ^b^Data missing in 2 recipients, ^c^ Data missing in 5 recipients, ^d^ Data missing in 7 recipients, ^e^ Data missing in 8 recipients, ^f^ Data missing in 9 recipients, ^g^ Data missing in 10 recipients, ^h^ Data missing in 11 recipients, ^i^ Data missing in 13 recipients, ^j^ Data missing in 14 recipients, ^k^ Data missing in 16 recipients. FPG and IbA1c values within 3 years of tie physical test date. *mGF*R, measured glomerular filtration rate; *PTI*, parathyroid hormone; *FP*G, fasting plasma glucose; *ESR*, erythrocyte sedimentation rate; *CRP*, c-reactive protein

All the kidney transplant recipients and controls attended school PE, and 18/24 recipients (75%) participated in a sports hobby at least once a week. Indexed physical performance test results of 24 pediatric kidney transplant recipients were compared between the transplant recipients attending only physical exercise (PE) at school and those attending PE and having at least one sports hobby per week. We did not find any significant difference in the test results according to self-reported physical activity.

### Physical performance in CNS and recipients with other primary diagnosis

Indexed physical test results of 24 pediatric kidney transplant recipients were divided in two diagnostic main groups: patients with congenital nephrosis (*n* = 15) and other primary diagnosis (*n* = 9). There was no significant difference between the groups, although the recipients with other diagnoses than CNS seemed to perform better in all test domains except for sit-and-reach and shuttle run. Children with CNS had a physical performance capacity of 4.13 (0.79) versus children with other CKD as primary diagnosis 4.48 (0.90), *p* = 0.174. Four recipients (26.6%) with CNS were identified as having delayed motor development during early childhood, which was resolved during the follow-up. When correlations of neurologic development and Physical Performance Total Capacity were examined, the correlations were not significant, but when a subgroup of early motor developmental delay was taken into account, the correlation with neurologic development and Physical Performance Total Capacity became significant (− 0.48, *p* = 0.02).

## Discussion

The present study on physical performance in pediatric kidney transplant recipients extends and confirms our previous finding that pediatric solid organ transplant recipients perform below the level of healthy controls in most of the test domains testing physical performance capacity*.* In leg lift and repeated squatting, pediatric kidney transplant recipients performed close to controls, suggesting that strength and endurance of lower body are less affected than strength and endurance of core muscles. This may be due to different factors, such as lower body muscle strength needed in activities of daily living, specific challenges to perform the test moves, and the effect of CKD in core muscle strength development during early childhood. Lower body strength and endurance are needed in many activities of daily living, such as walking up and down stairs, bending to put shoes on, and picking up things from the floor. Core-muscle strength was measured with specific test moves, which are not performed in everyday life, and needs to be specifically trained. In sit-and-reach, the recipients performed better than the controls, which may be due to muscle hypotonia related to CKD and also to slightly delayed puberty in many transplant recipients. Puberty affects the flexibility, especially in males [20].

In our patient cohort, the clinical parameters measuring kidney function, presence of metabolic disorders, or inflammation did not correlate with physical performance, which is contrary to the general presumption and our hypothesis. Moderate correlations were found with some clinical parameters, for example, height and sit-and-reach, DBP and sit-up test, FPG and shuttle run, and dialysis and shuttle run. We do not have an explanation to these correlations, and they appear to be quite random associations. It is of note that most of the clinical parameters in the transplant recipients were within normal range and GFR was relatively good, which has to be taken account when interpreting the results. This is one of the few prospective studies reporting physical performance in pediatric transplant recipients and the influence of clinical parameters upon it [21–25].

Our results showing lower physical performance capacity in kidney transplant recipients compared to healthy controls are consistent with previous findings [12, 26, 27]. The possible explanations for this finding may be diminished aerobic fitness, reduced VO_2_max, impaired peak VO_2_max, and reduced maximal heart rate [11, 21, 26, 28]. Furthermore, muscle strength below normative values in pediatric kidney transplant recipients has been reported by Krasnoff et al. [26] and Painter et al. [27]. Although the abnormalities in muscle metabolism seem to be corrected with a successful kidney transplantation [5], the decrease in aerobic fitness appears to persist into adulthood [22].

The relationship between kidney function and physical performance has remained controversial [10, 23]. CKD-related metabolic disorders, chronic inflammation, anemia, and malnutrion are generally accepted risk factors for frailty and attenuated physical performance [24, 25, 29, 30]. A recent study by Westphal Ladfors et al. [23] reported a significant correlation between GFR and exercise capacity in pediatric kidney transplant recipients, with low GFR predicting low exercise capacity. Our present study, however, did not find a correlation between GFR and exercise capacity, but the fact that mGFR was relatively good among all study subjects may have influenced our findings.

As well as the relationship between kidney function and physical performance, the correlation of different clinical parameters on physical performance in kidney transplant recipients has been uncertain in many studies. Sethna et al. [11] found that each 1-unit increase in hemoglobin was associated with a 5% greater VO_2_max, whereas Derakhshan et al. [10] found no such correlation between hemoglobin and VO_2_max. Watanabe et al. [31] found a positive correlation between hemoglobin and six-minute walk test (6MWT) distance. Our study did not find any significant correlation between physical performance capacity and hemoglobin, which may be at least partly due to the relatively high hemoglobin levels in all the study subjects. Takken et al. [32] and Watanabe et al. [31] found that hematocrit correlated positively with 6MWT distance, but hematocrit was not included in our analysis for assessing this correlation. Weaver et al. [13] found an inverse correlation between VO_2_max and serum creatinine, triglycerides, and C-reactive protein. In the present study, no significant correlation with physical performance and the aforementioned clinical parameters could be found. The effect of exercise on inflammation processes is of current and continuous interest in research of transplantation recipients following findings of moderate exercise producing inhibiting inflammatory cytokines and later beneficial effects on the immune system [33, 34]. The levels of inflammation markers were low in our study population, which might have influenced our results.

In our study cohort, patients with congenital nephrotic syndrome tended to perform worse than patients with other primary kidney disease. The difference between the groups was seen especially in tests measuring core muscle strength, i.e., sit-up and back extension, although the difference did not reach statistical significance. Similar findings of reduced core muscle strength were seen in our previous study concerning all pediatric solid organ transplant groups [2], which raises questions of why core muscle strength is especially affected after transplantation. Recipients with CNS have multiple possible explanations, including long-lasting hypoproteinemia, altered muscle structure regarding CKD [35] and sedentary lifestyle [36]. Of course, the question also remains whether the timing and modality of dialysis in recipients with CNS could have long-lasting effects on core muscle strength, since the treatment protocol consists of peritoneal dialysis starting at about the age of 6 months. The age of 6 months is an active time of development of motor skills in prone position and core control (development of prone, creeping, and on-all-four skills). Prone position or tummy time at least 30 min per day during waking hours appears beneficial for motor development [37]. In the present study, one third of the CNS patients had reportedly delayed motor development during their early life. Long-lasting hypoproteinemia before nephrectomy probably has a delaying effect on the development of motor skills in the early years, but on the other hand, recipients with CNS are transplanted at an early age (1.7 years vs. 5.4 years, *p* = 0.057). This could promote physical activity compared to recipients with non-transplanted CKD and therefore promote the achievement of motor skill milestones and better physical performance at older age. On the contrary, Wolf et al. [9] found that longer duration of CKD before transplant was not a significant risk factor for reduced physical activity in pediatric kidney transplant recipients, suggesting that even patients with a short period of CKD before transplantation struggle to achieve a normal level of physical activity after transplant. Our results suggest that testing physical performance even several years after kidney transplantation is warranted in this patient group.

Improving physical activity after pediatric kidney transplant and therefore promoting functional capacity and quality of life is important in improving the long-term outcomes of pediatric kidney transplant recipients. A Norwegian study reported that increasing early physiotherapy and follow-up of physical activity after pediatric kidney transplant had a significant positive impact on the cardiorespiratory fitness, quality of life, and mental health of pediatric kidney transplant recipients [38]. Then again, Carbonera et al. [39] found that home-based inspiratory muscle training in pediatric kidney transplant recipients did not significantly improve functional capacity or pulmonary function, but surprisingly, improved hemoglobin and hematocrit. The deficit in performance capacity of pediatric kidney transplant recipients seems undeniable, but the challenge of understanding the clinical markers affecting or predicting performance capacity remains. The means to improve physical capacity, reduce sedentary behavior and the impact of physical activity on clinical parameters warrant future studies.

### Study limitations

Despite being a nationwide study, the study sample remains small. This may have an impact on the study results. There is also an apparent limitation with interpreting the results of the shuttle run test, since problems with slipperiness of the floor prevented performing the test for a significant subset of patients (7/24). The slipperiness was a surprising problem with the floor material, even though the floor material in the physiotherapy unit should be suitable for physical performance testing. The problem could not be solved with indoor shoes or going barefoot. As for the clinical parameters and their associations with physical performance, some of the clinical parameters, such as GFR, hemoglobin, and infection markers, were at relatively good level in all study subjects, which might also have influenced our results (Table 4).

## Conclusions

The present study confirms earlier findings showing that physical performance of kidney transplant recipients is relatively good, but worse than that of healthy peers. The associations of clinical parameters and physical performance capacity warrant future studies to determine whether and how clinical parameters are associated with physical performance capacity. The reason for weakened core muscle strength also needs to be further studied. All pediatric kidney transplant recipients benefit from regular physiotherapy checkups as a routine treatment protocol to improve their physical capacity and to reduce sedentary lifestyle through counseling and encouragement. The patients with congenital nephrotic syndrome may require special monitoring of physical performance throughout childhood.

## Supplementary Information

Below is the link to the electronic supplementary material.

Graphical Abstract (PPTX 44 KB)

Supplementary file1 (DOCX 29.4 KB)

## Data Availability

The datasets generated and/or analyzed during the current study are available from the corresponding author on reasonable request.

## Abbreviations

CKD: Chronic kidney disease
BMI: Body mass index
CVD: Cardiovascular disease
GFR: Glomerular filtration rate
CNS: Congenital nephrotic syndrome of Finnish type
PE: Physical exercise
6MWT: Six-minute walk test

## References

[1] Jahnukainen T, Bjerre A, Larsson M, Tainio J (2016). The second report of the Nordic Pediatric Renal Transplantation Registry 1997–2012: more infant recipients and improved graft survivals. Pediatr Transplant.

[2] Mäenpää H, Tainio J, Jalanko H, Arokoski J, Jahnukainen T (2022). Physical performance after pediatric solid organ transplantation. Pediatr Transplant.

[3] Foster BJ, Kalkwarf HJ, Shults J, Zemel BS (2011). Association of chronic kidney disease with muscle deficits in children. J Am Soc Nephrol.

[4] Tsampalieros A, Kalkwarf HJ, Wetzsteon RJ, Shults J (2013). Changes in bone structure and the muscle-bone unit in children with chronic kidney disease. Kidney Int.

[5] Matsumoto N, Ichimura S, Hamaoka T, Osada T, Hattori M, Miyakawa S (2006). Impaired muscle oxygen metabolism in uremic children: improved after renal transplantation. Am J Kidney Dis.

[6] Endén K, Tainio J, Jalanko H, Jahnukainen K, Jahnukainen T (2018). Lower quality of life in young men after pediatric kidney transplantation when compared to healthy controls and survivors of childhood leukemia—a cross-sectional study. Transpl Int.

[7] Gustaw T, Schoo E, Barbalinardo C, Rodrigues N et al (2017) Physical activity in solid organ transplant recipients: participation, predictors, barriers, and facilitators. Clin Transplant 31(4). 10.1111/ctr.12929 10.1111/ctr.12929 28185297

[8] Kobashigawa J, Dadhania D, Bhorade S, Adey D (2019). Report from the American Society of Transplantation on frailty in solid organ transplantation. Am J Transplant.

[9] Wolf MF, George RP, Warshaw B, Wang E, Greenbaum LA (2016). Physical activity and kidney injury in pediatric and young adult kidney transplant recipients. J Pediatr.

[10] Derakhshan A, Derakhshan D, Amoozgar H, Shakiba MA, Basiratnia M, Fallahzadeh MH (2014). Exercise test in pediatric renal transplant recipients and its relationship with their cardiac function. Pediatr Transplant.

[11] Sethna CB, Salerno AE, McBride MG, Shults J (2009). Cardiorespiratory fitness in pediatric renal transplant recipients. Transplantation.

[12] Tangeraas T, Midtvedt K, Fredriksen PM, Cvancarova M, Mørkrid L, Bjerre A (2010). Cardiorespiratory fitness is a marker of cardiovascular health in renal transplanted children. Pediatr Nephrol.

[13] Weaver DJ, Kimball TR, Knilans T, Mays W (2008). Decreased maximal aerobic capacity in pediatric chronic kidney disease. J Am Soc Nephrol.

[14] Hovi L, Kurimo M, Taskinen M, Vettenranta J, Vettenranta K, Saarinen-Pihkala UM (2010). Suboptimal long-term physical performance in children and young adults after pediatric allo-SCT. Bone Marrow Transplant.

[15] Taskinen MH, Kurimo M, Kanerva J, Hovi L (2013). Physical performance of nontransplanted childhood ALL survivors is comparable to healthy controls. J Pediatr Hematol Oncol.

[16] Aho J, Häkkinen K, Kallinen M, Keskinen KL (2004) Kuntotestauksen käsikirja. Helsinki: Liikuntatieteellinen seura

[17] Tomkinson GR, Carver KD, Atkinson F, Daniell ND (2018). European normative values for physical fitness in children and adolescents aged 9–17 years: results from 2 779 165 Eurofit performances representing 30 countries. Br J Sports Med.

[18] Roufosse C, Simmonds N, Clahsen-van Groningen M, Haas M (2018). A 2018 reference guide to the Banff classification of renal allograft pathology. Transplantation.

[19] Flynn JT, Kaelber DC, Baker-Smith CM, Blowey D (2017). Clinical practice guideline for screening and management of high blood pressure in children and adolescents. Pediatrics.

[20] Kubo K, Kanehisa H, Kawakami Y, Fukanaga T (2001). Growth changes in the elastic properties of human tendon structures. Int J Sports Med.

[21] Weigmann-Faßbender S, Pfeil K, Betz T, Sander A (2020). Physical fitness and health-related quality of life in pediatric renal transplant recipients: an interventional trial with active video gaming. Pediatr Transplant.

[22] Tangeraas T, Midtvedt K, Cvancarova M, Hirth A (2011). Cardiorespiratory fitness in young adults with a history of renal transplantation in childhood. Pediatr Nephrol.

[23] Westphal Ladfors S, Bergdahl E, Hermannsson O, Kristjansson J (2021). Longitudinal follow-up on cardiopulmonary exercise capacity related to cardio-metabolic risk factors in children with renal transplants. Front Sports Act Living.

[24] Kaur K, Jun D, Grodstein E, Singer P (2018). Outcomes of underweight, overweight, and obese pediatric kidney transplant recipients. Pediatr Nephrol.

[25] Villasís-Keever MA, Zurita-Cruz JN, Serret-Montoya J, de Leon-Herrera AP (2021). Cardiometabolic factors in pediatric patients with chronic diseases. Arch Med Res.

[26] Krasnoff JB, Mathias R, Rosenthal P, Painter PL (2006). The comprehensive assessment of physical fitness in children following kidney and liver transplantation. Transplantation.

[27] Painter P, Krasnoff J, Mathias R (2007). Exercise capacity and physical fitness in pediatric dialysis and kidney transplant patients. Pediatr Nephrol.

[28] Lubrano R, Tancredi G, Falsaperla R, Elli M (2016). Cardiorespiratory fitness: a comparison between children with renal transplantation and children with congenital solitary functioning kidney. Ital J Pediatr.

[29] Atkinson MA, Martz K, Warady BA, Neu AM (2010). Risk for anemia in pediatric chronic kidney disease patients: a report of NAPRTCS. Pediatr Nephrol.

[30] Sylvestre LC, Fonseca KPD, Stinghen AEM, Pereira AM, Meneses RP, Pecoits-Filho R (2007). The malnutrition and inflammation axis in pediatric patients with chronic kidney disease. Pediatr Nephrol.

[31] Watanabe FT, Koch VH, Juliani RC, Cunha MT (2016). Six-minute walk test in children and adolescents with renal diseases: tolerance, reproducibility and comparison with healthy subjects. Clinics (Sao Paulo).

[32] Takken T, Engelbert R, van Bergen M, Groothoff J (2009). Six-minute walking test in children with ESRD: discrimination validity and construct validity. Pediatr Nephrol.

[33] Hemmati N, Kazemi S, Jamshidian-Tehrani N, Roozbeh J (2022). Effects of exercise training on immunological factors in kidney transplant recipients; a randomized controlled trial. Res Sports Med.

[34] Highton PJ, White AEM, Nixon DGD, Wilkinson TJ (2020). Influence of acute moderate- to high-intensity aerobic exercise on markers of immune function and microparticles in renal transplant recipients. Am J Physiol Renal Physiol.

[35] Alaylı G, Özkaya O, Bek K, Çalmaşur A (2008). Physical function, muscle strength and muscle mass in children on peritoneal dialysis. Pediatr Nephrol.

[36] Lui S, de Souza A, Sharma A, Fairbairn J (2020). Physical activity and its correlates in a pediatric solid-organ transplant population. Pediatr Transplant.

[37] Carson V, Lee E-Y, Hewitt L, Jennings C (2017). Systematic review of the relationships between physical activity and health indicators in the early years (0–4 years). BMC Public Health.

[38] Thorsteinsdottir H, Diseth TH, Lie A, Tangeraas T et al (2018) Small effort, high impact: focus on physical activity improves oxygen uptake (VO(2peak)), quality of life, and mental health after pediatric renal transplantation. Pediatr Transplant e13242. 10.1111/petr.13242 10.1111/petr.13242 29921004

[39] Carbonera RP, Barbosa APO, Normann TC, Lago PD, Garcia CD, Lukrafka JL (2020). Home-based inspiratory muscle training in pediatric patients after kidney transplantation: a randomized clinical trial. Pediatr Nephrol.

