# Supplementary material for: Physical performance capacity after pediatric kidney transplant and clinical parameters associated with physical performance capacity
Source: Pediatr Nephrol. 2022 Oct 31;38(5):1633–42. doi: 10.1007/s00467-022-05758-0 (PMC10060344; doi:10.1007/s00467-022-05758-0)
Supplement: Supplementary file 1 — Graphical Abstract (PPTX 44 KB) [file 467_2022_5758_MOESM1_ESM.pptx]

## Slide 1
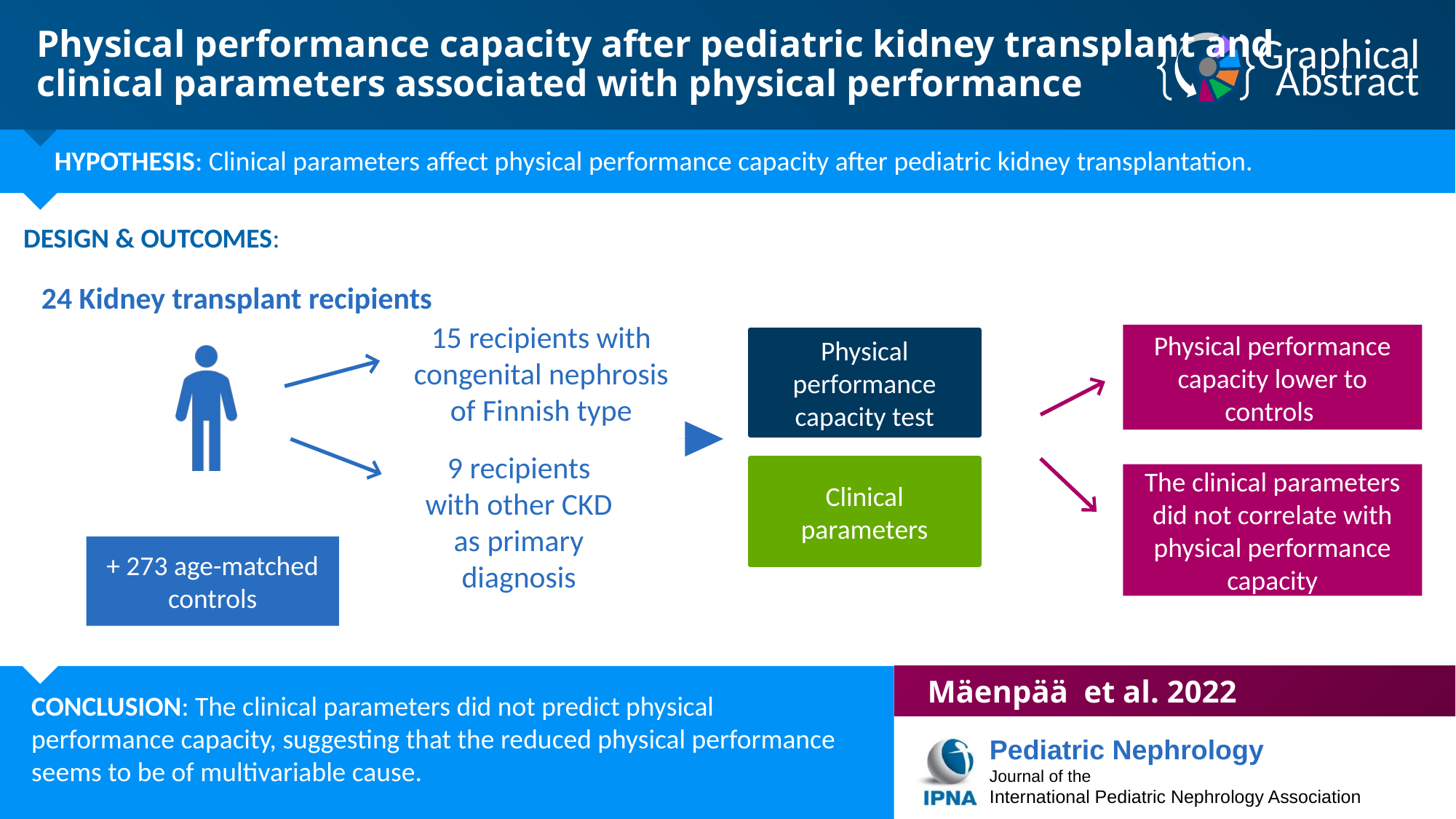

Physical performance capacity after pediatric kidney transplant and
clinical parameters associated with physical performance
HYPOTHESIS: Clinical parameters affect physical performance capacity after pediatric kidney transplantation.
DESIGN & OUTCOMES:
24 Kidney transplant recipients
15 recipients with congenital nephrosis of Finnish type
Physical performance capacity lower to controls
Physical performance capacity test
9 recipients with other CKD as primary diagnosis
Clinical parameters
The clinical parameters did not correlate with physical performance capacity
+ 273 age-matched controls
Mäenpää et al. 2022
CONCLUSION: The clinical parameters did not predict physical performance capacity, suggesting that the reduced physical performance seems to be of multivariable cause.
