# Supplementary material for: Physical performance capacity after pediatric kidney transplant and clinical parameters associated with physical performance capacity
Source: Pediatr Nephrol. 2022 Oct 31;38(5):1633–42. doi: 10.1007/s00467-022-05758-0 (PMC10060344; doi:10.1007/s00467-022-05758-0)
Supplement: Supplementary file 2 — Supplementary file1 (DOCX 29.4 KB) [file 467_2022_5758_MOESM2_ESM.docx]

| Appendix 1. Correlation coefficient of indexed physical test results and recipients characteristics in 24 pediatric kidney transplant recipients | | | | | | | | | | | |  |  |
| --- | --- | --- | --- | --- | --- | --- | --- | --- | --- | --- | --- | --- | --- |
|  | Leg lift | | | Repeated squatting | | Sit-up | | Sit-and-reach | | Back extension | | Shuttle run | |
|  | rₛ | | p-value | rₛ | p-value | rₛ | p-value | rₛ | p-value | rₛ | p-value | rₛ | p-value |
| Median age at the time of testing | 0.26 | | 0.23 | 0.07 | 0.76 | 0.13 | 0.55 | –0.27 | 0.20 | 0.15 | 0.49 | –0.05 ⁷ | 0.84 |
| Median age at the time of transplant | 0.03 | | 0.88 | 0.06 | 0.78 | –0.13 | 0.53 | –0.12 | 0.56 | 0.10 | 0.66 | –0.46 ⁷ | 0.06 |
| Height | –0.03 | | 0.88 | –0.19 | 0.38 | 0.06 | 0.78 | –0.43 | 0.04* | 0.02 | 0.92 | 0.05 ⁷ | 0.84 |
| Weight | –0.01 | | 0.97 | –0.12 | 0.58 | –0.06 | 0.80 | –0.32 | 0.13 | –0.11 | 0.63 | –0.01 ⁷ | 0.96 |
| BMI | 0.05 | | 0.83 | 0.09 | 0.68 | –0.19 | 0.37 | –0.08 | 0.72 | –0.04 | 0.86 | –0.09 ⁷ | 0.74 |
| Systolic blood pressure (mmHg) | 0.00 | | 0.99 | 0.01 ² | 0.96 | 0.29 ² | 0.19 | 0.20 ² | 0.36 | 0.38 ² | 0.08 | –0.22 ⁸ | 0.42 |
| Diastolic blood pressure (mmHg) | 0.21 | | 0.36 | 0.13 ² | 0.58 | 0.67 ² | 0.00* | 0.36 ² | 0.10 | 0.31 ² | 0.16 | 0.28 ⁸ | 0.29 |
| Number of rejections | 0.04 | | 0.86 | 0.02 | 0.94 | –0.19 | 0.38 | 0.16 | 0.47 | 0.05 | 0.83 | –0.17 ⁷ | 0.52 |
| Dialysis duration | –0.47 | | 0.02* | –0.24 | 0.27 | –0.37 | 0.08 | 0.19 | 0.38 | –0.21 | 0.32 | 0.31 | 0.23 |
| Dialysis treatment modality | 0.14 | | 0.51 | 0.06 | 0.80 | 0.11 | 0.62 | 0.22 | 0.32 | 0.16 | 0.48 | 0.26⁸ | 0.31 |
| Neurology (normal/deviant) | –0.57 | | 0.00* | –0.49 | 0.02* | –0.25 | 0.23 | 0.32 | 0.12 | –0.34 | 0.10 | 0.57 | 0.02* |
| Psychic challenges (normal/deviant) | 0.34 | | 0.10 | –0.04 | 0.86 | –0.12 | 0.58 | –0.26 | 0.22 | –0.22 | 0.31 | –0.47 ⁷ | 0.06 |
| Laboratory results |  | |  |  |  |  |  |  |  |  |  |  |  |
| mGFR (mL/min/1.73 m²) | 0.20 | | 0.36 | 0.13 | 0.55 | 0.11 | 0.60 | –0.12 | 0.56 | 0.04 | 0.84 | 0.15 ⁷ | 0.56 |
| Creatinine (umol/L) | 0.01 | | 0.95 | –0.04 | 0.84 | 0.18 | 0.39 | 0.03 | 0.91 | 0.11 | 0.62 | 0.11 ⁷ | 0.67 |
| Cystatin C (mg/L) | –0.06 | | 0.77 | 0.02 | 0.91 | –0.05 | 0.81 | 0.31 | 0.14 | 0.11 | 0.61 | 0.04 ⁷ | 0.87 |
| Urea (mmol/L) | –0.31 | | 0.14 | –0.33 | 0.12 | –0.22 | 0.30 | 0.09 | 0.68 | –0.26 | 0.22 | –0.16 ⁷ | 0.55 |
| Hemoglobin (g/L) | –0.02 | | 0.92 | 0.18 | 0.39 | 0.17 | 0.43 | –0.13 | 0.54 | 0.34 | 0.11 | –0.16 ⁷ | 0.54 |
| Urine protein (mg/L) | –0.42 ⁵ | | 0.08 | 0.39 ⁵ | 0.10 | 0.07 ⁵ | 0.79 | 0.06 ⁵ | 0.79 | –0.01 ⁵ | 0.97 | –0.45 ¹º | 0.11 |
| Potassium (mmol/L) | –0.16 | | 0.45 | –0.18 | 0.40 | –0.46 | 0.03* | 0.12 | 0.58 | –0.30 | 0.15 | –0.07 ⁷ | 0.79 |
| Sodium (mmol/L) | 0.03 | | 0.89 | –0.26 | 0.21 | –0.26 | 0.22 | –0.15 | 0.50 | –0.25 | 0.24 | 0.22 ⁷ | 0.40 |
| Phosphate (mmol/L) | 0.10 | | 0.64 | 0.19 | 0.38 | –0.26 | 0.22 | 0.08 | 0.70 | 0.19 | 0.38 | 0.05 ⁷ | 0.86 |
| Magnesium (mmol/L) | 0.33 | | 0.12 | 0.33 | 0.12 | 0.11 | 0.60 | 0.35 | 0.09 | 0.56 | 0.00* | 0.18 ⁷ | 0.50 |
| Calcium-ionized (mmol/L/pH 7.4) | –0.16 | | 0.46 | –0.34 | 0.10 | 0.02 | 0.92 | –0.18 | 0.40 | –0.17 | 0.43 | –0.27 ⁷ | 0.29 |
| PTH (ng/L) | 0.11 ¹ | | 0.63 | 0.03 ¹ | 0.91 | 0.34 ¹ | 0.12 | 0.15 ¹ | 0.51 | 0.31 ¹ | 0.15 | –0.00 ⁸ | 1.00 |
| FPG (mmol/L) | –0.22 ¹³ | | 0.53 | –0.01 ¹³ | 0.98 | –0.35 ¹³ | 0.29 | –0.11 ¹³ | 0.74 | –0.21 ¹³ | 0.54 | 0.69 ¹¹ | 0.04* |
| HbA1c (mmol/mmol) | –0.04 ¹⁴ | | 0.90 | 0.49 ¹⁴ | 0.15 | –0.41 ¹⁴ | 0.24 | 0.20 ¹⁴ | 0.58 | –0.28 ¹⁴ | 0.43 | –0.11 ¹⁶ | 0.80 |
| Vitamin D | –0.03 ¹ | | 0.88 | –0.11 ¹ | 0.63 | –0.15 ¹ | 0.49 | –0.12 ¹ | 0.60 | –0.30 ¹ | 0.17 | 0.15 ⁸ | 0.57 |
| Triglyceride (mmol/L) | 0.08 | | 0.74 | –0.23 ² | 0.30 | 0.04 ² | 0.86 | –0.22 ² | 0.32 | –0.03 ² | 0.88 | –0.41 ⁹ | 0.13 |
| ESR (mm/h) | –0.05 ² | | 0.82 | –0.01 ² | 0.98 | –0.16 ² | 0.47 | –0.21 ² | 0.34 | 0.27 ² | 0.22 | 0.01 ⁸ | 0.98 |
| CRP (mg/L) | –0.02 ² | | 0.95 | 0.18 | 0.40 | 0.29 | 0.18 | 0.29 | 0.17 | 0.21 | 0.33 | 0.40 ⁷ | 0.12 |
| rₛ=Spearman´s correlation coefficient was used, p-value two-tailed, ¹ Data missing in 1 recipient ² Data missing in 2 recipients, ⁵ Data missing in 5 recipients, ⁷ Data missing in 7 recipients, ⁸ Data missing in 8 recipients, ⁹ Data missing in 9 recipients ¹º Data missing in 10 recipients. FPG and HbA1c values within 3 years of the physical test date. mGFR, measured glomerular filtration rate; PTH, parathyroid hormone; FPG, fasting plasma glucose; ESR, erythrocyte sedimentation rate; CRP, c-reactive protein | | | | | | | | | | | | | |
|  | |  |  |  |  |  |  |  |  |  |  |  |  |
|  | |  |  |  |  |  |  |  |  |  |  |  |  |
